# Signs of a turning tide in social norms and attitudes toward abortion in Ethiopia: Findings from a qualitative study in four regions

**DOI:** 10.1186/s12978-021-01240-6

**Published:** 2022-06-13

**Authors:** Kathryn A. O’Connell, Addisalem T. Kebede, Bereket M. Menna, Mengistu T. Woldetensay, Sara E. Fischer, Ghazaleh Samandari, Jemal K. Kassaw

**Affiliations:** 1 EngenderHealth, District of Columbia, 505 Ninth Street NW, Suite 601, Washington, DC 20004 USA; 2 EngenderHealth Ethiopia, Djibouti Avenue, P.O. Box 156 code 1110, Addis Ababa, Ethiopia; 3 Georgetown University, 3700 O St. NW, Washington, DC 20007 USA; 4 University of North Carolina, Chapel Hill, NC 27599-2200 USA

**Keywords:** Abortion, Safe abortion, Ethiopia, Barriers, Stigma, Attitudes, Pregnancy

## Abstract

**Background:**

Despite the 2005 expansion in abortion legal indications in Ethiopia, which provided for abortions in cases of rape, incest, or fetal impairment and other circumstances, nearly half of abortions occurred outside health facilities in 2014. The purpose of this study is to explore and understand the social barriers women face in seeking and obtaining quality safe abortion care, as a means to generate evidence that could be used to improve access to and quality of abortion services.

**Methods:**

Thirty-two focus group discussions with both men and women were held in four different regions of Ethiopia: Addis Ababa; Amhara; Oromia; and the Southern Nations, Nationalities, and Peoples’ Region. The study team recruited participants (n = 193) aged 18–55 in each region using a purposive sample with snowball recruitment techniques. We conducted discussions in Amharic or Afaan Oromo using a semi-structured guide and transcribed and translated them into English for analysis. We used deductive coding and analysis to categorize findings into emergent themes around stigma, barriers, and the changing nature of attitudes around abortion.

**Results:**

Despite changes in abortion law, findings show that women with unwanted pregnancies and those seeking abortions are still heavily stigmatized and sanctioned in a number of communities across Ethiopia. Abortion was deemed unacceptable in most cases, though respondents were more tolerant in cases of risk to the mother’s life and of rape. We saw promising indications that changes are taking place in Ethiopian society’s view of abortion, and several participants indicated progress toward a more supportive environment overall for women seeking abortion care. Still, this progress may be limited by variable knowledge of abortion laws and tightly held gender-based social norms, particularly in rural areas. Most participants noted the importance of education and outreach to improve abortion attitudes and norms.

**Conclusion:**

Policymakers should create further awareness in Ethiopia on the availability of quality abortion services in public health facilities and the indications for legal abortion. Such efforts should be based on principles of gender equality, as a means of ensuring enduring changes for women’s reproductive choice throughout the country.

**Supplementary Information:**

The online version contains supplementary material available at 10.1186/s12978-021-01240-6.

## Plain language summary

We conducted a study to explore and understand the social barriers women face in seeking and obtaining quality safe abortion care in Ethiopia. We implemented focus group discussions with both men and women in four different regions of Ethiopia. We analyzed our findings into themes around stigma, barriers, and the changing nature of attitudes around abortion. Our results show that women seeking abortions are still heavily stigmatized. We found that abortion was deemed unacceptable in most cases. However, we also saw promising indications that changes are taking place in Ethiopian society’s view of abortion, and several participants indicated progress toward a more supportive environment overall for women seeking abortion care. Still, this progress may be limited by variable knowledge of abortion laws. Most participants noted the importance of education and outreach to improve abortion attitudes and norms. We concluded that further awareness is needed in Ethiopia on the availability of quality abortion services in public health facilities and the indications for legal abortion. These efforts should consider gender equality to ensure enduring changes for women’s reproductive choice throughout the country.

**Supplementary Information:**

The online version contains supplementary material available at 10.1186/s12978-021-01240-6.

## Background

Globally, an estimated 99 million unintended pregnancies occur each year and Ethiopia’s estimated unintended pregnancy rate is notable [1]. Although data are variable according to geographical variations and study methodologies, estimates are that 13.7 to 36.5% of all Ethiopian pregnancies are unintended [2–4]. Furthermore, several studies have found that sociodemographic factors, including marital status (e.g., being formerly married, never married, or living alone), may be associated with increased likelihood of unintended pregnancies [5]. A woman’s age also contributes to the likelihood of her experiencing an unintended pregnancy. In Ethiopia, national estimates are that 10% of adolescents ages 15–19 have given birth, with several notable variations. For example, adolescents in rural areas are three times more likely to have begun childbearing than their urban peers and the percentage of adolescents who have begun childbearing rises from 3% among those with more than a secondary education to 12% among those with a primary education and 28% among those with no education [6].

Unintended pregnancy arises from obstacles to family planning services, including abortion. Stigma against sexual activity among unmarried adolescents and young women remains an obstacle to Ethiopian women accessing these services [7]. Moreover, among the many barriers to safe abortion care (SAC) is abortion stigma*.* Stigma need not be visible for it to have a detrimental effect on decision processes, and abortion stigma is itself a “social phenomenon” that is constructed locally [8]. At the core, stigma represents an unequal access to power and resources that is reinforced through institutions and systems [8]. Stigma therefore manifests in social relationships and cultural constructs, and little is known about the particular manifestations and pathways of abortion stigma on women seeking SAC services in Ethiopia. Silence and fear of social exclusion can have a profound effect on the willingness of women to speak openly about reproductive health needs and family planning decisions [8]. Prescribed gender roles and male dominance in society underpin the stigma faced by women regarding family planning decisions [9].

In Ethiopia, negative attitudes toward unintended pregnancy, particularly for young, unmarried women, create an isolating environment because premarital sex and pregnancy are seen as shameful and inappropriate [10, 11]. Unmarried pregnant women are often socially excluded, rejected by friends and families, and must make difficult decisions to cope with the physical and psychological repercussions of her pregnancy [10, 11]. For example, it has been shown that most adolescents do not speak to their families about sexual health concerns [12], and students who become pregnant often have a solitary decision-making experience [10]. In contrast to attitudes about unintended pregnancy, attitudes about abortion in Ethiopia seem to be shifting. In recent surveys, only around half of respondents reported disagreeing with abortion legalization and having generally negative attitudes toward abortion [13, 14]; more people do agree with abortion in particular cases, such risk to health, rape, or incest. Interestingly, women with greater knowledge of the abortion law have been shown to have more favorable views about abortion [15].

In 2005, Ethiopia expanded its abortion law to allow termination in cases of rape, incest, or fetal impairment. In addition, a woman can legally terminate a pregnancy if her life or physical health is in danger, if she has a physical or mental health disability, or if she is a minor who is physically or mentally unprepared for childbirth [16]. An estimated 620,300 induced abortions were performed in Ethiopia in 2014, resulting in an annual abortion rate of 28 per 1000 women ages 15–49; of these, an estimated 47% (294,100 abortions) occurred outside of health facilities [17]. Evidence suggests that a significant number of women do not seek care for abortion-related complications [18], and women continue to use unsafe methods to induce abortions outside of health facilities [19]. Adolescents may be particularly at risk, given their increased likelihood to access illegal services outside the health system, resulting in increased abortion-related complications [9]. As such, even in settings where abortion services are legalized, women may continue to use unsafe abortion services [9].

Despite the relative liberalization and expansion of abortion services in Ethiopia, studies have shown several barriers to accessing abortion services, including gaps in knowledge of where to obtain services, negative perceptions of the cost, concerns about privacy and confidentiality, fear of provider judgment, negative societal attitudes toward abortion, obsolete facilities, and weak referral systems [9, 20–22]. Barriers to accessing SAC services are further exacerbated by confusion about the legality of abortion and application of the law at the local level [9], the knowledge of which remains relatively low despite the law being 15 years old [23, 24].

In addition, recent quantitative evidence from Addis Ababa demonstrates several provider barriers, including their lack of understanding of specific provisions of the law [25]. However, nearly three-fourths of the providers were not comfortable working in a site where pregnancy termination was performed, and only one-fourth of participants agreed with providing legal abortion under any circumstances [21, 26]. In addition, a recent qualitative study with Ethiopian health providers revealed mixed perceptions about providing abortion services. For some, religious norms and the belief that the early fetus has a moral right to life served as a barrier toward the provision of these services. For other providers, the acknowledgment of the interests and needs of a pregnant woman supported their provision of such services. In short, providers differed in their perceptions and held different values. To the authors' knowledge, no prior studies have assessed attitudes about abortion providers among the Ethiopia population, nor included qualitative methods to address community perceptions around why providers may be unwilling to provide this service in Ethiopia.

Although the liberalization of Ethiopia’s abortion law reduces policy restrictions on abortion, the inescapable hold of stigma as a social construct continues to play a role in decision making as well as access to quality SAC services. The purpose of this study is to explore the social context within which abortion occurs and to understand the current attitudes that contribute to women’s accessing timely and quality abortion care in Ethiopia.

## Methods

This study uses qualitative focus group discussions (FGDs) to explore people’s understanding of and attitudes around abortion phenomena in their communities. This method is particularly appropriate when seeking to understand social norms and attitudes that influence behaviors of individuals within specific communities [27].

### Instrument

All FGDs used a semi-structured interview guide that was translated into local dialects and back-translated for verification. The interview guide included questions on perceptions of what women do when they experience an unintended pregnancy and why they may experience unintended pregnancy, community reactions to their pregnancy, awareness of the abortion law in Ethiopia, and perceptions of women who have terminated their pregnancy.

Prior to data collection, the instrument was pre-tested with four respondents that were not included in the main study. After the pre-test, researchers discussed the findings and further refined the instrument.

### Study setting

Ethiopia is the oldest independent country in Africa, with the second largest population after Nigeria. The country is divided into nine ethnic regional states and two administered cities. Listed in order of largest population to smallest, the regions are Oromia; Amhara; Southern Nations, Nationalities, and Peoples’ (SNNP); Somali; Tigray; Afar; Benishangul Gumuz; Gambella; and Harari [28]. Oromia, Amhara, and SNNP together comprise slightly more than 80% of the total population [31]. The two city administrations are Addis Ababa and Dire Dawa, which hold 3.7 and 0.5% of the total country population, respectively. Although Ethiopia is a country with more than 80 ethnic groups, the Oromo and Amhara people together account for more than 60% of the population (34.5 and 26.9%, respectively) [28].

Data collection took place across three regions of Ethiopia: Amhara; Oromia; and Southern Nations, Nationalities, and Peoples’ Region (SNNPR) and one administrative city: Addis Ababa. These areas represent a diverse mix of Ethiopians, ranging in rural–urban, economic and educational statuses. Given the qualitative nature of this formative research study, the design calls for a representative and exhaustive consideration of the individual differences across these areas. We considered urban, peri-urban, and rural settings, as well as areas where there were higher and lower rates of abortions in these areas.

### Participants and recruitment

We discussed details of the study with community leaders in each region, and then data collectors collaborated with local health extension workers to identify a range of respondents. Once initial recruits were identified, a snowball sampling method was used. Participants were recruited from different zones within the region and then invited to join FGDs from other zonal areas in order to ensure privacy and confidentiality. None of the participants were known to the FGD moderators or note takers. To capture a wide range of social attitudes toward abortion, the study recruited both male and female participants and divided groups by age: a “younger” group ages 18 to 29 versus an “older” group ages 30 to 55. Groups were also segmented by the participants’ gender.

### Focus group discussions

Data collection took place between June and July of 2018. Eight FGDs were conducted in each region, ensuring a balanced distribution of age and gender groupings, with 32 FGDs overall. Each FGD had six participants (one had seven), and the average age of participants for the younger FGDs was 23 years and for the older FGDs was 37 years. All individuals were welcome to participate, regardless of prior knowledge or experience with abortion. In total, 193 individuals participated, of which 96 were female and 56.5% were from urban areas (Table 1).

**Table 1:** Study participant demographics

	N (%)
Gender	
Male	97 (50.3)
Female	96 (49.7)
Age	
18–29	103 (53.4)
30 +	90 (46.6)
Location	
Urban	109 (56.5)
Rural	84 (43.5)
Total	193

### Data collection

Teams of eight Ethiopian social scientists (four women and four men) were trained to use the FGD guides and study protocols for data management during a four-day training and served either as moderators or note takers. Female moderators conducted the FGDs with female participants. FGDs were held in community centres.

Prior to participation, researchers informed study participants of the study objectives and confidentiality measures. Verbal consent was obtained from all participants. No incentives were provided. The FGDs took around 90 min, including a 10–15 min break.

FGDs were conducted in Amharic or Afaan Oromo. Researchers made voice recordings of all FGDs, with the consent of participants, and interviewers also took notes of the content, nonverbal behavior, and setting of the interaction.

### Analysis

Recordings from all interviews were transcribed verbatim in Amharic or Afaan Oromo and then translated into English. On completion, a member of the bilingual research team read and reviewed each translated English transcript, comparing it against the original Amharic or Afaan Oromo version. The team rectified any discrepancies with the translator until full agreement between the translated transcript and original Amharic or Afaan Oromo version was obtained.

Transcripts were initially coded inductively, with key words or phrases extracted verbatim from the discussion. Using the extracted codes from the initial transcript, a codebook with rules was created to ensure that all coding was consistent across the 32 FGDs. Where a new code emerged in future transcripts, it was either coded together with an existing code (if the meanings were synonymous) or a new code was created. Occasionally, a code was discarded during the second phase if it proved to be an idiosyncratic response. Ensuring that all responses were initially coded uniformly resulted in a data-driven coding manual that facilitated the later thematic analysis [29].

Based on the codes generated by the first analysis phase, we used deductive coding [30] and analysis to categorize findings into emergent themes around stigma, barriers, and the changing nature of attitudes around abortion. Researchers analyzed the frequency with which individuals reported themes, and to what extent the group mentioned these themes, for patterns. This procedure helped us clarify which themes consistently emerged across all groups and which were idiosyncratic. We then organized statements according to key themes related to unintended pregnancy and abortion.

Other data analysts conducted a further review of 60% of the transcripts. Through this comparison, the team checked to make sure members had coded the themes in a consistent manner, without creating new codes in one type of analysis and not the other. Any discrepancies were resolved through discussion with the larger team of coders and the primary analyst until full agreement was obtained.

The lead researchers also presented the results to the Ethiopia research team upon completing the analysis, but not to community members. In addition, the team once again verified any quotes used in the results summary with the original transcripts.

## Results

Our findings suggest that although abortion is stigmatized and social norms remain largely negative, some signs of a shift in attitudes toward abortion surfaced during our discussions. Several main themes emerged from the FGDs: negative attitudes toward unplanned, premarital pregnancy remain strong; attitudes about abortion are shifting to include consideration of the context in which the pregnancy took place; and the perception of abortion providers is variable. First, we will present the evidence on persistent negative attitudes around unplanned pregnancy and abortion; next, we will present our findings on how attitudes around abortion are slowly changing; finally, we will discuss some of the divided perceptions of the abortion law and abortion providers.

### Norms of stigma, shame, and rejection

#### Prevailing negative attitudes around unplanned pregnancy

An important antecedent to abortion for many women and girls in the study communities was the stigma and social backlash of unplanned pregnancy, mainly for young, unmarried women and girls. Across the various urban and rural communities where this study took place, the resulting behaviors toward unplanned pregnancy is one of extreme rejection, judgment, and isolation. Slightly less than half of participants mentioned the society’s negative opinion of unplanned pregnancy—mostly attributed to younger age (e.g., being a student and occurring before marriage); lack of awareness or use of family planning methods; or rape—and almost three-quarters mentioned either a negative opinion or subsequent life problems. No respondent mentioned that unplanned pregnancy for an unmarried woman would result in a positive societal perception (Additional file 1).

*When a pregnancy occurs to a woman who isn’t married or is a student, the community considers this to be very shameful, as well as terrifying for the woman and disappointing to the family and kin group. Female, Oromia, age 22*

*[I]f the unintended pregnancy occurs out of marriage or before marriage, it will be a big problem for the girl. She will be worried as a result and shamed by her community and relatives. Male, Addis Ababa, age 21*

*The society won’t help her, they will only point their fingers at her. They will try to solve the puzzle of who the father might be, they will spread the gossip to every corner they can find. If she can deal with this, she will have a baby in her arms. But the society’s reaction might throw her off, so she might consider abortion. Male, Hawassa, age 21*

Participants commonly expressed how this stigma included outright denunciation and ridicule of the girl by community members. Participants discussed whether she was seen as a “good girl” or a “bad girl” in the minds of community members. Many respondents confirmed that a girl would be seen as bad or could even damage a previously good reputation with an unintended pregnancy.

*People will consider her unintended pregnancy as resulting from her behavioral problem. People will consider her as a slut. People will see her as a bad girl, as promiscuous. They will not have love for her and see her as not a good girl. Male, Bole, age 27*

*If she was considered as a good girl, if she has a good behavior in the eye of the community, and if she experiences unintended pregnancy and abortion, people’s opinion about her will change. They will no more consider her as a good girl. They will not see her as they used to. Female, Tirunesh, age 19*

Very often, respondents mentioned that girls would be used as cautionary tales or made examples of in the community to warn others of the perils of unintended pregnancy.

*The people would hate her, or would dislike even to see her, and they wouldn’t want to approach her. The society would mainly think that she will teach this bad thing to others, and hence they would instruct their children to stay far from her, and due to such awful connotation on her, her future life would be distorted wherein she could be unable to get a marriage partner. This is because, it is mainly assumed that she has destroyed a life. Male, Oromia, age 30*

Harsh societal reaction against unplanned pregnancies among unmarried women or girls was commonly perceived as resulting in devastating outcomes for the pregnant woman. As mentioned above, many people described life problems that would extend beyond societal pressures like rejection or isolation. Discussants frequently recalled stories of depression, poverty, stopping education, homelessness, excommunication, and even suicide among girls who had been rejected because of an unplanned pregnancy. This stigma was described as following a girl into her future, putting her prospects of marriage and social cohesion at risk.

*In the case of most adolescent students, if a girl encountered unintended pregnancy and her families knew that, she could be hated and discriminated. This may cause her to flee away from home and in these situations, she may deliver her baby in the streets, may lose her education as well as her life dreams. Not only being separated from her family and relatives, even the guy who impregnated her may not want to accept her and for this reason, she may suffer a lot. Female, Oromia, age 21*

Even in cases where the family may initially support the girl, community shaming would add considerable pressure on families to reject their own daughters. Many participants relayed stories of families that sent their pregnant daughters away to avoid the humiliation brought on by the unplanned pregnancy. Others mentioned that families might force the girl to hide and secretly have an abortion to preserve the family’s reputation.

*In my opinion it is not just about a family accepting the girl who has unintended pregnancy or not, it is mainly about what the community will feel and think about the girl. Families will not hate their daughter even though she has unintended pregnancy. But they will think about how they will be perceived if the members of the community know that the girl is pregnant. They will consider her pregnancy as a shame and disrespect. In our culture girls are expected to be married before having children. Otherwise, it is considered as disrespect. Female, Meshualekia, age 19*

#### Persisting stigma around abortion

After participants discussed unplanned pregnancies, the discussion turned to the issue of abortion. Discussants were first asked what people in their community would think about a woman who had an abortion. Well over half of the participants responded that abortion would be considered in a negative light, with no qualifications (Additional file 1). No women in the younger age category thought that support would be given to a woman who had terminated a pregnancy. In these cases, a common perception across all participants was the belief that women and girls who abort their pregnancies were considered to be sinful, murderous, and not deserving of respect.

Several participants claimed they would not support a woman who chose to have an abortion and that such a woman would be looked down upon as compared to one who had never had an abortion. Here, as with those who had an unplanned pregnancy, women and girls may face stigma and isolation by their choice to terminate a pregnancy. Such stigma could also hinder a woman’s future, particularly her “marriageability” in the eyes of her community.

*No [the community] won’t support [a woman who wants an abortion] at all. They even try to point fingers on her because…it is considered as she took life or committed a murder. There is a character of saying, “she took a life and who on earth is going to help or support her!?” Even there are people who don’t want her to even sit beside them. Female, Amhara, age 23*

*I haven’t seen people supporting a woman who had abortion after unintended pregnancy. In my opinion, I guess when abortion is done, a life is lost. It is difficult to ignore that. And people also accept that what is right is to be careful not to be pregnant initially. But to abort the fetus after the woman gets pregnant is unacceptable by many people. Male, Hiwot amba, age 32*

The data suggest that this rejection of abortion and stigmatization of women may stem from a deeply religious culture that believes life is a sacred gift from God, whose plan must prevail over the preferences of women. So strong is this belief among some participants that they would actively encourage or pressure a woman to keep her pregnancy if she were considering abortion. For some, the sin of pregnancy termination was too great to justify abortion and would only compound the original offense of being pregnant outside of marriage.

*Firstly, that is a mistake in God’s eyes; it is not allowed to abort a pregnancy in our religions. In addition, that child, secondly, can also grow up to be a person of many impacts on the society. Therefore, I will advise her to change her idea of aborting her pregnancy. I will try to get her to give birth to the child. I will do that. Not just for a family member, but that is also what I will do for a neighbor or a community member. Female, Hiwot amba, age 44*

*I will pressure her to have the baby. To have abortion is to make a mistake twice. The first mistake is when she had the unintended pregnancy. That is also a sin in God’s eyes. And in addition to have the abortion is also a mistake. It is like to stop a life. That is also a mistake and a sin. Therefore, even though she had committed the first mistake by having the unintended pregnancy, there is no need to make the second mistake. Therefore, I will not agree with her wanting to have an abortion. Male, Meshualekia, 24*

Some participants, though a minority, do not believe that abortion should be allowed under any circumstances. Even in the case of rape, for example, these individuals may see it as a duty to bear the child and not to compound the event with “murder” of the fetus.

*Even if it is like that, I will not advise her to have an abortion. It can be unintentional or rape, but it is better for her to raise the kid. Since she is going to take a human life, I will tell her to give birth to it. Female, Amhara, age 27*

Interestingly, an oft-cited reason for not supporting abortion was that the child may grow up to be an important person in the country, like a leader or ruler. This idea cut across respondents and made it clear that for many in Ethiopia, any fetus should be treated with great respect.

*There are people who are incapable of conceiving a child; a child is a gift from God. To abort a child—meaning to end a life—is a major sin. This child might turn out to be a very important person for the world. However, due to the couple’s reason he cannot experience life at all. Male, SNNPR, age unknown*

### Turning tides and mixed views on abortion

As mentioned above, most of the initial reactions to abortion were negative. However, our results show that attitudes are not as unforgiving as they first seem. Notably, for all questions regarding abortion, there were a significant number of responses exhibiting mixed views—mainly, that positions depended upon the circumstances surrounding the abortion (Additional file 2). This suggests that when prompted, most participants did not actually see abortion as a black or white issue but were willing to consider conditions under which abortion would be appropriate or even preferred. Thus, despite the presence of abortion stigma among the participants, many community members also recognized recent, positive societal shifts in perceptions around abortion.

For example, many discussants expressed support for abortion for rape survivors, but a number of those expressed the need to “verify” the rape before offering acceptance or support. Responses from discussants suggested an underlying suspicion of women who claimed rape in order to abort and that sufficient evidence of the rape must be provided in order to validate a woman’s assertion and decision to abort. If such verification were provided, they felt that the woman would be supported.

*For a girl that was raped and pregnant from the rape I believe it should be allowed. Abortion should be allowed for her. But it has to be based on reason. If there is evidence that indicates that she was raped, then she should be allowed to have the abortion. But it should not be allowed without evidence indicating that she was raped. The evidence should indicate that the girl was physically forced and raped and not just was out there having alcohol and taking drugs. Female, Tirunesh, age 19*

*In such cases, the society would support her. Where there is tangible evidence particularly, for instance where she has shouted for help, the people would not sleep until those responsible for the rape were brought to justice in addition to providing her with all the necessary support. As such, there is a due emphasis from the society on such issues. Male, Oromia, age 40*

Likewise, although some participants implied that rape in the context of “bad behavior” would not be a satisfactory cause for acceptable abortion, others suggested that such a perception of so-called bad girls or good girls would determine the community’s reaction: those previously seen as good by the community would not be blamed for an unintended pregnancy.

*I think the reaction of the community will depend on the situation; it depends on the behavior of the girl and how the pregnancy happened. If the girl was considered as a good girl and as a girl that respects her parents and people in the neighborhood, then even if she has unintended pregnancy the community may not have a bad attitude towards her. But if she is a girl that falls out of line and if she used to show desires to engage in unacceptable behavior, then people will blame her and talk bad things about her. Female, Meshualekia, age 21*

Therefore, many of the views around abortion were nuanced, suggesting that societal reactions would often depend upon context. This finding was particularly clear when discussants were asked how they would react if the person who wanted the abortion was a family member. Although the majority suggested that they would still prefer for the family member to give birth or would somehow try to convince her not to have an abortion—even helping her financially if her reason behind wanting the abortion was poverty—many also agreed that this would depend upon the situation. Particularly in cases of incest, rape, or health risk, many suggested that they would support her in her decision to terminate a pregnancy.

*My decision will depend on what a doctor will say about the pregnancy. I will also ask her why she wanted to have the abortion. If she wanted to have the abortion because of economic reasons, then I will not allow her to have the abortion. I will do everything to support her. But if it is because of health reasons, then I will agree with the abortion if it can be safely done. Male, Meshualekia, age 19*

### Perception of support around abortion

As described above, societal perspectives on abortion were not simply negative, and many reactions depended upon particular qualifications (e.g., proof of rape or prior opinion of the girl within the community). This demonstrates Ethiopians’ willingness to consider the circumstances under which an abortion might take place. However, these were not the only responses that indicated a shift in attitudes toward abortion, and many responses—particularly under certain conditions—were positive, even without such qualifications.

#### Acceptability of abortion in case of health risk or rape

Encouragingly, when asked about particular conditions—rape and risk to the mother’s life—most discussants were far more supportive than about abortion more generally. For example, there were five times more supportive responses when participants were asked about abortions conducted when pregnancy was a result of rape, and eight times more supportive responses (i.e., more than three-quarters of responses) when participants were asked about abortion to save the mother’s life. Additionally, although gender and age group did not result in significant differences on level of support, respondents from urban areas suggested societal support for abortion in the cases of rape or health threat much more often than did those from rural areas (Additional file 2).

Many participants recognized the difficulty that a raped woman might face in bearing and raising the child borne out of violence and would offer support to her if she made that choice.

*A woman who performed an abortion due to rape will receive better acceptance and better treatment among the society than the others who normally perform abortion. The society will be very understanding and will try to make the girl feel much better by giving her different life advice. Even family will be very supportive throughout the process, telling her they will be there for her. Female, SNNPR, age 25*

Furthermore, discussants viewed abortion due to health risk to be out of the woman’s control and recognized it as a tragic necessity rather than an act of volition. Furthermore, they commonly acknowledged that the mother’s life was to be protected, even if it was at the expense of the fetus. In these situations, women would be treated with compassion and understanding rather than with the judgment or derision reserved for those who abort because of personal choice. Indeed, participants shared that women facing this type of abortion would be treated just as a woman who had given birth, with family and community members visiting her and supporting her in the post-abortion period. One discussant even shared her own experience following an abortion due to a high-risk pregnancy, reporting feeling very cared for by her community members.

*I have gone through the same experience some years ago. I was forced to go through abortion because doctors told me that the pregnancy would be dangerous for me. They referred me to Dinberua Hospital and I had to go through the process. And everyone in the community showed me care and support. They all came and visited me. I haven’t experienced any form of judgment or anything that I consider to be negative. Female, Bole, age 33*

#### Increase in unqualified support for abortion

Even beyond such qualified (albeit positive) responses, an emerging theme among discussants was the perceived overall increase in the number of people capable of understanding a woman’s or girl’s choice to abort regardless of circumstance and that many of them may even provide material or moral support. This support was given in spite of the prevailing norms against abortion and in the face of stigma and pressure from others in the society. There was also evidence from participants to suggest that in certain communities there would be no negative reaction to a woman who chooses to terminate a pregnancy. This finding was seen as an important change from previous times and was largely attributed to an increased education among communities about abortion.

*From the general trend that prevails in neighborhood or society or the country as a whole, [abortion] is something disgusting, an act that shouldn’t be thought of and that would drive a hostile reaction from the majority. However, there are a few people who think that it has already happened and who would show sympathy for her and provides support no matter what adverse attitude from others in the society could be there. Male, Oromia, age 30*

*There is no negative reaction to the girl today. Today people are educated and negative thinking and attitude towards abortion are nonexistent. In the past it was a big problem. Now people have awareness about the causes for the occurrence of unintended pregnancy. Male, Hiwot amba, 40*

### Variable knowledge of Ethiopian abortion law

After eliciting perspectives on abortion under various circumstances, participants were asked to recount their understanding of the legality of abortion in Ethiopia. The resulting responses revealed a muddled notion of legal indications of abortion in the country, with no notable differences according to the age, gender, or location of the participant. Knowledge of the Ethiopian abortion law varied widely, from those who believed it was not permissible under any circumstance (approximately one-third of responses) to those who recalled that it was allowed in some cases (e.g., rape, risk to the mother’s health, or incest; slightly fewer than half of responses).^1^One-quarter of discussants openly admitted to not knowing the Ethiopian abortion law at all (Additional file 3). Some participants even interpreted the question to mean legal repercussions of abortion and were adamant that a woman would be prosecuted in the event of an abortion.

*The law in Ethiopia doesn’t allow abortion. This is so because, since the aborted baby [the fetus in the womb] has a life and could be raised to become a man/woman, and for this reason, [abortion] is considered as a murder and is thus a criminal act. Female, Oromia, age 23*

*I think there is a law about abortion because the girl might be raped forcefully, her body might not able to carry the fetus, or the pregnancy might endanger her health. Therefore, I think there might be a law allowing abortion. Male, Amhara, age 20*

### Diverging views on abortion providers

Just as they held variable views on stigma against abortion, participants held conflicting opinions on the role of abortion providers. The responses to this question were evenly distributed across those who perceived them negatively, those who thought they were helping, and those who were either unsure or agreed that the societal perspective would be mixed (Additional file 3). For those who were opposed to abortion, providers were seen as an enabler of that which should not be permitted. This was particularly true of providers that offer abortion to women who were not in immediate danger. For some participants, abortion providers were seen as working actively against the will of God.

*I personally view them as sinners. That is because they are participating in abortion and abortion is seen as stopping a life. Female, Amhara, age 37*

*Whatever their reason [for the abortion], the community sees only whether abortion is done or not. If it is done, they believe that [the provider] is destroying the building of God. Even, they will say if this kid is born, it may help the community when it grows up and also they will say it is the wish of God for this kid to come to the world but [the provider] terminated it. Therefore, the feeling is different, and it is not a good feeling that the community have. Male, Amhara, age 26*

For others, legal abortion providers were seen as providing a helpful service, or at least just doing their job. However, participants often indicated that those who perform abortions illegally or with herbs, such as traditional healers, were condemned because such abortions can be very dangerous for the woman.

*Abortion is done legally and illegally. Those who work legally are accepted and there is nothing against these people. But those who work illegally in the community are not accepted. People don’t have a good opinion on these people. Male, Hiwot Amba, age 32*

On the other hand, a common theme to emerge from participants was the view that abortion providers offered essential services to save or protect women’s lives. These discussants understood that abortion providers were supplying necessary assistance according to the laws of the country and viewed them as benefitting the community. For some, abortion providers were considered God-like (an interesting contrast to the responses above) and making an indispensable contribution to society.

*Instead of dying due to giving birth at a young age, it is a good deed if a doctor can abort the baby to save her life. For these exceptions, they are helpful and necessary; we should be thankful. We don’t know what the baby will turn out to be or what problem it will bring to the society and his mother…. If they refuse to help, she might suffer several problems to the point of ending her life. Their existence, the profession itself is necessary for the community. Female, SNNPR, 20*

*The people have good perception and adore the health professionals that help women to have the abortion; and it is even said “waaqa biraa doktaratuu jiraa” [literally to mean that a doctor is another God]. Male, Oromia, age 30*

## Discussion

Using data from 32 focus group discussions across Ethiopia, this qualitative study adds important information to our understanding of abortion attitudes and behaviors in the country. In particular, the study demonstrates the expectations and assumptions underlying abortion stigma against women in this society and highlights the changing nature of acceptability of abortion in Ethiopia. The study found that starting from the onset of unintended pregnancy, women in Ethiopia (particularly young and unmarried women) are highly stigmatized and isolated. Both male and female participants in all age ranges and regardless of level of rurality shared that when unmarried women experience pregnancy, they are stigmatized and often driven to depression and sometimes homelessness, poverty, or suicide because of the social backlash they receive, including rejection, isolation, and gossip. On the other hand, very few respondents mentioned of men’s role in these incidents, laying the social burden of unintended pregnancy on women and female adolescents alone. This type of stigma can drive women to seek unsafe abortions and further threaten their social standing and lives [31, 32].

Although there was near-unanimous rejection of unplanned pregnancy among study discussants, the topic of abortion evinced more complex responses; although stigma was still present, many participants expressed a growing openness and acceptance of abortion. The majority of participants felt that unless the termination was due to serious reasons, such as pregnancy resulting from incest or to protect the health of the mother, the act would be considered “sinful” or “murderous” and women who chose to terminate a pregnancy would be rejected by their families and their communities. Furthermore, even in cases of rape, participants were not always supportive of women seeking abortion services. In these instances, the circumstances of the rape itself and the “quality” of the woman sometimes came into play; if the woman was perceived to have put herself in a position to be raped, there was little mercy and she was blamed for the rape. Here, the evidence suggests a bias against women who choose to live their lives outside of prescribed gender norms, which can result in denunciation of the woman and her choices [33]. In Ethiopia, young, unmarried women in particular are forced to navigate a minefield of social pressure in order to terminate a pregnancy and thus make up the majority of those seeking clandestine, unsafe abortions across the country [34, 35].

Though common themes showed negative beliefs around abortion, other emerging themes suggested that stigma around abortion in Ethiopia may be softening. Discussants reported a change in overall support, with more families or communities starting to provide emotional, financial, and practical support for abortion clients; a few participants even went so far as to disclaim any discrimination against them in their local communities, suggesting an evolution in tolerance for abortion. Furthermore, the majority of discussants suggested that through improved education and outreach efforts, communities would increase their understanding and support for women seeking abortion.

Our study also found divergent views about the role of providers that may offer abortion services. Around one-third of participants saw abortion providers in Ethiopia as healers supporting and assisting women, whereas an additional one-third saw them as sinners acting out against God. Indeed, these findings also highlight the role of stigma and how it manifests on an interpersonal level in the interaction between women seeking abortion services and providers. Provider attitudes are among the most commonly cited barriers to reproductive and sexual health services for adolescents described in the literature [8]. For example, data of providers’ attitudes about abortion reveal complex underlying forces such as training, professional norms, and religious beliefs as contributors to the willingness of providers to provide abortion services [21]. This is true of not only providers but other health facility staff that may come into contact with a woman seeking to terminate an unintended pregnancy [36]. Thus, past and present interactions with providers can serve as either facilitators or barriers to abortion services.

Our results can also be situated in the context of several interventions that are underway by development partners in Ethiopia, in collaboration with the Ministry of Health, to improve access to quality safe abortion care. The context includes efforts to develop and disseminate evidence-based national standards and guidelines in 2006 [37] and a revision in 2014 to incorporate recent World Health Organizations recommendations [38]: training health professionals at all levels of the health system with a particular focus on task-shifting to midlevel providers; integrating safe abortion and post-abortion contraception into existing reproductive health services; building the capacity of Primary Health Care Units to sustain comprehensive abortion care services; and engaging with the private sector to expand its capacity to provide safe abortion care services. In addition, other nonprofit organizations support providing comprehensive post-abortion care services as well as safe induced abortion for all legal indications (as allowed by national law). Nonprofit organizations have been implementing a variety of interventions to raise awareness about abortion care issues to reach a wide range of audiences living in both urban and rural areas. As such, some of the positive shifts in attitudes among our respondents are perhaps reflective of such interventions that have aimed to improve the quality of services and information about access to safe abortion services.

This study should be viewed in light of a few limitations. First, the samples for the FGDs were purposive, with recruits being suggested by health workers and through a snowball method of recruitment. Findings may not be representative of overall community perceptions. Next, although the nature of FGDs allowed for participants to have a natural conversation around a particular topic, there is the possibility for intragroup response bias, whereby one or two people might respond in a certain way and the remaining participants simply agree. Still, while this sort of effect is not entirely preventable, a number of people felt comfortable disagreeing with the majority opinion. Furthermore, the nature of qualitative data limits generalizability of findings beyond the specific subgroups interviewed here. However, great care was taken to recruit a diverse range of FGD participants across several regions in Ethiopia to provide as complete a picture of abortion attitudes as possible. Finally, although we made efforts to recruit participants from different regions, genders, ages, and ethnicities to better address the research objectives, the results of the analysis did not identify large differentiations according to these different segments.

## Conclusion

This study reveals deeply ingrained negative and gender-biased social norms against unintended pregnancy and abortion in Ethiopia, a stigma that is particularly pronounced for young or unmarried women. These biases can affect women’s reproductive choices and limit their ability to access timely and safe abortion care as they negotiate their desire to terminate pregnancy with the real and often extreme personal costs of revealing an abortion to family and community members. Nevertheless, findings suggest pockets of growth in the acceptability of abortion in Ethiopia, progress that may be scaled and cemented with appropriate educational and social behavior change campaigns. Implementors and policymakers should create further awareness in Ethiopia of the legality of abortion, the mental and physical repercussions of stigma and social rejection, and the importance of supporting women to access safe and timely abortion care. Such efforts should be based on principles of gender equality, as a means of ensuring enduring changes for women’s reproductive choice across the country. By improving knowledge and attitudes around abortion across the country, Ethiopians can begin to provide an enabling environment of support and care for all women seeking abortion.

## Supplementary Information

**Additional file 1.** Negative attitudes around unintended pregnancy.

**Additional file 2.** Turning tides and mixed views around abortion.

**Additional file 3.** Knowledge of abortion law and perception of abortion providers.

## Data Availability

The data that support the findings of this study are available from EngenderHealth but restrictions apply to the availability of these data, which were used under license for the current study, and so are not publicly available. Data are, however, available from the authors upon reasonable request and with permission of EngenderHealth.

## Abbreviations

FGD: Focus group discussion
SAC: Safe abortion care
SNNPR: Southern Nations Nationalities and Peoples Region

## Footnotes

1 As of 2005 in Ethiopia, abortion is allowed in the case of rape, incest, fetal impairment, physical danger to the woman, physical or mental impairment of the woman, or physical/mental inadequacy of a minor [Ethiopia Ministry of Health, Health Sector Development Program IV in Line with GTP, 2010/11–2014/15, Addis Ababa, Ethiopia: Federal Democratic Republic of Ethiopia, 2010].
