# Supplementary material for: Signs of a turning tide in social norms and attitudes toward abortion in Ethiopia: Findings from a qualitative study in four regions
Source: Reprod Health. 2022 Jun 13;19(Suppl 1):198. doi: 10.1186/s12978-021-01240-6 (PMC9195190; doi:10.1186/s12978-021-01240-6)
Supplement: Supplementary file 1 — Additional file 1. Negative attitudes around unintended pregnancy. [file 12978_2021_1240_MOESM1_ESM.docx]

**Supplementary File 1**

Appendix Table 1^§^

|  | Why do women experience unwanted pregnancies? | What happens when women experience unwanted pregnancies? | What would people in your community think about Meseret*? |
| --- | --- | --- | --- |
| **GRAND TOTAL** | Rape = 54 | Negative opinion = 105 | Negative Reaction = 100 |
|  | Lack of Awareness / Contraception = 76 | Abortion = 55 | Support = 12 |
|  | Pressure = 84 | Life Problems = 74 | Mixed Reaction = 63 |
| **TOTAL MALE** | Rape = 25 | Negative opinion = 46 | Negative Reaction = 59 |
|  | Lack of Awareness / Contraception = 49 | Abort = 29 | Support = 7 |
|  | Pressure = 48 | Problems = 47 | Mixed Reaction = 28 |
| **TOTAL FEMALE** | Rape = 29 | Negative opinion = 59 | Negative Reaction = 41 |
|  | Lack of Awareness / Contraception = 27 | Abort = 26 | Support = 5 |
|  | Pressure = 36 | Problems = 27 | Mixed Reaction = 35 |
| **TOTAL 18-29** | Rape = 32 | Negative opinion = 61 | Negative Reaction = 56 |
|  | Lack of Awareness / Contraception = 43 | Abort = 20 | Support = 3 |
|  | Pressure = 47 | Problems = 39 | Mixed Reaction = 37 |
| **TOTAL 30+** | Rape = 22 | Negative opinion = 44 | Negative Reaction = 44 |
|  | Lack of Awareness / Contraception = 33 | Abort = 34 | Support = 12 |
|  | Pressure = 37 | Problems = 35 | Mixed Reaction = 26 |
| **TOTAL URBAN** | Rape = 30 | Negative opinion = 62 | Negative Reaction = 53 |
|  | Lack of Awareness / Contraception = 44 | Abort = 25 | Support = 8 |
|  | Pressure = 42 | Problems = 41 | Mixed Reaction = 39 |
| **TOTAL RURAL** | Rape = 24 | Negative opinion = 43 | Negative Reaction = 47 |
|  | Lack of Awareness / Contraception = 32 | Abort = 30 | Support = 4 |
|  | Pressure = 34 | Problems = 32 | Mixed Reaction = 24 |

§ The numbers in this table refer to the number of times a response (or part of a response) was coded in a particular way. As is often the case in FGDs, some people would provide multi-part answers that were coded in more than one way for a single question, whereas other times some discussants would not respond to a question at all. Therefore, the numbers should not be regarded as absolutes but rather can only represent the broader trends in responses and volume of mentions within a particular category.

*At the beginning of each FGD, participants were shown a photo of a young woman and were told that she had undergone abortion. This fictional person was used as an example throughout the discussion to ask questions about how she would be perceived.
