# Supplementary material for: Signs of a turning tide in social norms and attitudes toward abortion in Ethiopia: Findings from a qualitative study in four regions
Source: Reprod Health. 2022 Jun 13;19(Suppl 1):198. doi: 10.1186/s12978-021-01240-6 (PMC9195190; doi:10.1186/s12978-021-01240-6)
Supplement: Supplementary file 2 — Additional file 2. Turning tides and mixed views around abortion. [file 12978_2021_1240_MOESM2_ESM.docx]

**Supplementary File 2**

Appendix Table 2^§^

|  | Would people in your community accept Meseret’s* action and support her? Or would they isolate her or make her feel negatively about the abortion? | Would the reaction to Meseret’s having an abortion be different if her pregnancy was the result of being raped? What would be different? | What would your reaction be to Meseret’s having an abortion if her pregnancy presented a risk to her life? What would be different? | If a family member came to you wishing to terminate a pregnancy, how do you think you would respond? |
| --- | --- | --- | --- | --- |
| **GRAND TOTAL** | Most people support = 11 | Most people support = 59 | Most people support = 88 | Stop the abortion = 103 |
|  | Most people don’t support = 85 | Most people don’t support = 41 | Most people don’t support = 6 | Support her = 31 |
|  | Mixed Reaction = 40 | Mixed Reaction = 63 | Mixed Reaction = 20 | Mixed Reaction = 58 |
| **TOTAL MALE** | Most people support = 6 | Most people support = 29 | Most people support = 47 | Stop the abortion = 46 |
|  | Most people don’t support = 45 | Most people don’t support = 22 | Most people don’t support = 2 | Support her = 22 |
|  | Mixed Reaction = 21 | Mixed Reaction = 32 | Mixed Reaction = 10 | Mixed Reaction = 29 |
| **TOTAL FEMALE** | Most people support = 5 | Most people support = 30 | Most people support = 41 | Stop the abortion = 57 |
|  | Most people don’t support = 40 | Most people don’t support = 19 | Most people don’t support = 4 | Support her = 9 |
|  | Mixed Reaction = 19 | Mixed Reaction = 31 | Mixed Reaction = 10 | Mixed Reaction = 29 |
| **TOTAL 18-29** | Most people support = 3 | Most people support = 27 | Most people support = 47 | Stop the abortion = 51 |
|  | Most people don’t support = 54 | Most people don’t support = 28 | Most people don’t support = 6 | Support her = 16 |
|  | Mixed Reaction = 23 | Mixed Reaction = 41 | Mixed Reaction = 12 | Mixed Reaction = 43 |
| **TOTAL 30+** | Most people support = 8 | Most people support = 32 | Most people support = 41 | Stop the abortion = 52 |
|  | Most people don’t support = 31 | Most people don’t support = 17 | Most people don’t support = 0 | Support her = 15 |
|  | Mixed Reaction = 17 | Mixed Reaction = 22 | Mixed Reaction = 8 | Mixed Reaction = 15 |
| **TOTAL URBAN** | Most people support = 6 | Most people support = 39 | Most people support = 58 | Stop the abortion = 57 |
|  | Most people don’t support = 49 | Most people don’t support = 18 | Most people don’t support = 3 | Support her = 20 |
|  | Mixed Reaction = 26 | Mixed Reaction = 36 | Mixed Reaction = 10 | Mixed Reaction = 35 |
| **TOTAL RURAL** | Most people support = 5 | Most people support = 20 | Most people support = 30 | Stop the abortion = 46 |
|  | Most people don’t support = 36 | Most people don’t support = 23 | Most people don’t support = 3 | Support her = 11 |
|  | Mixed Reaction = 14 | Mixed Reaction = 27 | Mixed Reaction = 10 | Mixed Reaction = 23 |

§ The numbers in this table refer to the number of times a response (or part of a response) was coded in a particular way. As is often the case in FGDs, some people would provide multi-part answers that were coded in more than one way for a single question, whereas other times some discussants would not respond to a question at all. Therefore, the numbers should not be regarded as absolutes but rather can only represent the broader trends in responses and volume of mentions within a particular category.

* At the beginning of each FGD, participants were shown a photo of a young woman and were told that she had undergone abortion. This fictional person was used as an example throughout the discussion to ask questions about how she would be perceived.
