# Supplementary material for: Signs of a turning tide in social norms and attitudes toward abortion in Ethiopia: Findings from a qualitative study in four regions
Source: Reprod Health. 2022 Jun 13;19(Suppl 1):198. doi: 10.1186/s12978-021-01240-6 (PMC9195190; doi:10.1186/s12978-021-01240-6)
Supplement: Supplementary file 3 — Additional file 3. Knowledge of abortion law and perception of abortion providers. [file 12978_2021_1240_MOESM3_ESM.docx]

**Supplementary File 3**

Appendix Table 3^§^

|  | What is the abortion law in Ethiopia? | What do people say about the person (doctor, nurse, midwife) who have helped the women have the abortion? |
| --- | --- | --- |
| **GRAND TOTAL** | Abortion is prohibited = 46 | Negative Reaction = 41 |
|  | I don't know = 33 | Positive or Acceptable Reaction = 42 |
|  | Abortion is allowed in some cases = 61 | Mixed Reaction / Unsure = 43 |
| **TOTAL MALE** | Abortion is prohibited = 18 | Negative Reaction = 19 |
|  | I don't know = 14 | Positive or Acceptable Reaction = 24 |
|  | Abortion is allowed in some cases = 35 | Mixed Reaction / Unsure = 24 |
| **TOTAL FEMALE** | Abortion is prohibited = 28 | Negative Reaction = 22 |
|  | I don't know = 19 | Positive or Acceptable Reaction = 18 |
|  | Abortion is allowed in some cases = 26 | Mixed Reaction / Unsure = 19 |
| **TOTAL 18-29** | Abortion is prohibited = 25 | Negative Reaction = 18 |
|  | I don't know = 19 | Positive or Acceptable Reaction = 21 |
|  | Abortion is allowed in some cases = 37 | Mixed Reaction / Unsure = 33 |
| **TOTAL 30+** | Abortion is prohibited = 21 | Negative Reaction = 23 |
|  | I don't know = 14 | Positive or Acceptable Reaction = 21 |
|  | Abortion is allowed in some cases = 24 | Mixed Reaction / Unsure = 10 |
| **TOTAL URBAN** | Abortion is prohibited = 28 | Negative Reaction = 22 |
|  | I don't know = 19 | Positive or Acceptable Reaction = 27 |
|  | Abortion is allowed in some cases = 40 | Mixed Reaction / Unsure = 28 |
| **TOTAL RURAL** | Abortion is prohibited = 18 | Negative Reaction = 19 |
|  | I don't know = 14 | Positive or Acceptable Reaction = 14 |
|  | Abortion is allowed in some cases = 21 | Mixed Reaction / Unsure = 16 |

§ The numbers in this table refer to the number of times a response (or part of a response) was coded in a particular way. As is often the case in FGDs, some people would provide multi-part answers that were coded in more than one way for a single question, whereas other times some discussants would not respond to a question at all. Therefore, the numbers should not be regarded as absolutes but rather can only represent the broader trends in responses and volume of mentions within a particular category.
